# Gray and white matter differences in the medial temporal lobe in late-life depression: a multimodal PET-MRI investigation

**DOI:** 10.1017/S0033291724003362

**Published:** 2025-02-04

**Authors:** Akihiro Takamiya, Ahmed Radwan, Daan Christiaens, Margot Van Cauwenberge, Thomas Vande Casteele, Maarten Laroy, Kristof Vansteelandt, Stefan Sunaert, Michel Koole, Jan Van den Stock, Koen Van Laere, Filip Bouckaert, Mathieu Vandenbulcke, Louise Emsell

**Affiliations:** 1 Department of Neurosciences, Neuropsychiatry, KU Leuven, Leuven, Belgium; 2 Department of Neurosciences, KU Leuven, Leuven Brain Institute, Leuven, Belgium; 3 Hills Joint Research Laboratory for Future Preventive Medicine and Wellness, Keio University School of Medicine, Tokyo Japan; 4 Department of Imaging and Pathology, Translational MRI, KU Leuven, Leuven, Belgium; 5 Department of Electrical Engineering, EST-PSI, KU Leuven, Leuven, Belgium; 6 Department of Neurosciences, Research Group Psychiatry, Neuropsychiatry, Academic Center for ECT and Neuromodulation (AcCENT), University Psychiatric Center KU Leuven, Kortenberg, Belgium; 7 Department of Radiology, University Hospitals Leuven, Leuven, Belgium; 8 Department of Imaging and Pathology, Nuclear Medicine and Molecular Imaging, KU Leuven, Leuven, Belgium; 9 Department of Geriatric Psychiatry, University Psychiatric Center KU Leuven, Leuven, Belgium; 10 Department of Nuclear Medicine and Molecular Imaging, University Hospitals Leuven, Leuven, Belgium

**Keywords:** diffusion MRI, late-life depression, medial temporal lobe, PET, Tau, vascular depression

## Abstract

**Background:**

Late-life depression (LLD) is characterized by medial temporal lobe (MTL) abnormalities. Although gray matter (GM) and white matter (WM) differences in LLD have been reported, few studies have investigated them concurrently. Moreover, the impact of aetiological factors, such as neurodegenerative and cerebrovascular burden, on tissue differences remains elusive.

**Methods:**

This prospective cross-sectional study involved 72 participants, including 33 patients with LLD (mean age 72.2 years, 23 female) and 39 healthy controls (HCs) (mean age 70.6 years, 24 female), who underwent clinical and positron emission tomography (PET)-magnetic resonance imaging (MRI) assessments. High-resolution 3D T1-weighted and T2-weighted FLAIR images were used to assess MTL GM volumes and white matter hyperintensities (WMHs), a proxy for cerebrovascular burden. Diffusion kurtosis imaging metrics derived from multishell diffusion MRI data were analyzed to assess WM microstructure in the following MTL bundles reconstructed using constrained spherical deconvolution tractography: uncinate fasciculus, fornix, and cingulum. Standardized uptake value ratio of 18F-MK-6240 in the MTL was used to assess Alzheimer’s disease (AD) type tau accumulation as a proxy for neurodegenerative burden.

**Results:**

Compared to HCs, patients with LLD showed significantly lower bilateral MTL volumes and WM microstructural differences primarily in the uncinate fasciculi bilaterally and right fornix. In patients with LLD, higher vascular burden, but not tau, was associated with lower MTL volume and more pronounced WM differences.

**Conclusions:**

LLD was associated with both GM and WM differences in the MTL. Cerebrovascular disease, rather than AD type tau-mediated neurodegenerative processes, may contribute to brain tissue differences in LLD.

## Introduction

Late-life depression (LLD: older individuals with major depressive disorder [MDD]) is associated with both GM and white matter (WM) differences (Kim & Han, 2021). LLD has been associated with lower GM volume in frontolimbic circuits involved in emotion regulation and cognitive processing, especially in the medial and lateral prefrontal cortex, orbitofrontal cortex, anterior cingulate cortex, and subcortical brain regions (Kim & Han, 2021; Sexton et al., 2013; Zhukovsky et al., 2021). Among them, lower volumes of the medial temporal lobe (MTL), including the hippocampus, have been reported frequently and consistently (De Winter et al., 2017; Du et al., 2014; Geerlings and Gerristen, 2017; Sexton et al., 2013; Taylor et al., 2020). In addition, WM microstructural differences, detected on diffusion-weighted imaging (DWI), have also been reported, yet the number of studies is much smaller than those investigating GM (Kim & Han, 2021; Wen et al., 2014). Although a previous meta-analysis reported an association between LLD and differences in the uncinate fasciculus, the number of included studies was small (study *N* = 3 including a total of 67 patients with LLD) (Wen et al., 2014). Furthermore, a fundamental issue with prior DWI research on LLD is the utilization of diffusion tensor imaging (DTI)-derived metrics, including fractional anisotropy (FA). Despite its widespread use, DTI has several limitations due to the confounding effect of intravoxel WM fiber dispersion (Jeurissen et al., 2013; Pasternak et al., 2018). More advanced approaches such as diffusion kurtosis imaging (DKI), which quantifies the amount of non-Gaussian diffusion and constrained spherical deconvolution (CSD), which is able to model crossing fibers, provide more reliable assessments of WM microstructure (e.g. by differential sensitivity to intra/extracellular diffusion and improved fiber bundle reconstruction, respectively).

Investigations into the etiology of GM and WM differences in LLD have suggested two hypotheses: ‘accelerated’ and ‘pathological’ brain aging (Szymkowicz et al., 2023). The ‘accelerated’ brain aging model describes a phenomenon where normal biological aging occurs more rapidly than typically expected, with biological characteristics appearing older than an individual’s actual chronological age (Christman et al., 2020). One example of this model is cerebrovascular burden, a common feature in aging (Szymkowicz et al., 2023). Cerebrovascular disease has been proposed as a key factor in LLD pathophysiology (Taylor et al., 2013). Supporting this notion, a meta-analysis found increased white matter hyperintensities (WMHs), a proxy for cerebral small-vessel disease (SVD), in patients with LLD (van Agtmaal et al., 2017). Additionally, such cerebrovascular burden in patients with LLD has been associated with long-term negative clinical outcomes, including greater cognitive decline (e.g., executive dysfunction) (Butters et al., 2008), and poorer responses to pharmacological treatments (Sheline et al., 2010; Szymkowicz et al., 2023).

The ‘pathological’ aging process includes amyloid and/or tau accumulation (Szymkowicz et al., 2023). This model assumes a similar pathological neurodegenerative process in LLD to neurodegenerative diseases, such as Alzheimer’s disease (AD). This hypothesis is supported by epidemiological data linking LLD with an elevated risk of developing dementia (Da Silva et al., 2013; Diniz et al., 2013). Neuropathological (Rapp et al., 2008; Sweet et al., 2004) and *in vivo* positron emission tomography (PET) studies (Babulal et al., 2016; Donovan et al., 2018; Harrington et al., 2017) have reported an association between depressive symptoms and increased amyloid accumulation. However, these studies primarily focused on healthy individuals or dementia patients with depressive symptoms or a history of depression, rather than on patients with a psychiatric diagnosis of LLD. In contrast, recent large-scale *in vivo* PET studies specifically focusing on LLD as a primary psychiatric disorder did not find any evidence of increased amyloid accumulation (De Winter et al., 2017; Mackin et al., 2021; Takamiya et al., 2021). Additionally, the results from two studies investigating tau accumulation in patients with LLD have been inconsistent (Moriguchi et al., 2021; Vande Casteele et al., 2022), possibly due to differences in PET tracers and clinical characteristics of the participants.

Overall, the two most consistent neuroimaging findings of LLD are lower GM volumes in the MTL region and increased cerebrovascular burden represented as increased WMH volumes. However, it is still unclear how these two major findings are linked. Furthermore, no study has applied advanced DWI modeling in the LLD population for the assessment of WM alterations, and there is no consensus on which WM bundles are involved in the neurobiology of LLD. Furthermore, no studies have explored the combined impact of potential aetiological factors, such as cerebrovascular burden and pathological protein accumulation, on GM/WM differences in LLD.

In this study, we conducted a comprehensive analysis of multimodal PET-MRI data to gain insight into the underlying etiology and the associated GM and WM changes in LLD. First, we investigated GM differences in the MTL region, as well as microstructural differences in WM fiber bundles connected to the MTL, including the uncinate fasciculus, fornix, and cingulum bundle, in patients with LLD. To overcome the limitations of DTI modeling (Jeurissen et al., 2013; Pasternak et al., 2018), we analyzed multishell diffusion MRI, incorporating advanced techniques such as CSD-based tractography and DKI. Second, we explored potential aetiological factors underlying the GM and WM differences, using WMH volume as a proxy for cerebrovascular-related brain aging, and tau accumulation as a proxy for a ‘pathological’ brain aging process (Szymkowicz et al., 2023).

## Methods

### Study participants

Clinical and multimodal neuroimaging data were collected from participants in Leuven Late-Life Depression (L3D) study (Emsell et al., 2021). This study was approved by the ethics committee of the University Hospitals Leuven (S61968) and was conducted at KU Leuven. All participants provided written informed consent prior to enrollment in the study in accordance with the Declaration of Helsinki. Patients with LLD were recruited at the University Psychiatric Center KU Leuven, Belgium. Inclusion criteria for the LLD group include the following: (1) age over 60 years old; (2) a diagnosis of MDD according to the Diagnostic and Statistical Manual of Mental Disorders (DSM)-5. Exclusion criteria include the following: (1) primary referral for assessment of cognitive impairment; (2) comorbid major psychiatric illness; (3) a diagnosis of neurological disease (e.g., AD, Parkinson’s disease, or stroke); (4) past or current alcohol or drug abuse; (5) any contraindication for MRI scanning. Healthy controls (HCs) were recruited from the local community by distributing posters, flyers, and information brochures. Inclusion/exclusion criteria for HC were the same as the LLD, except for a diagnosis of MDD.

### Clinical assessment

Geriatric Depression Scale (GDS, 30 items) and Montgomery-Asberg Depression Rating Scale (MADRS) were used to assess depression severity, and the Mini-Mental State Examination (MMSE) was used to assess global cognitive function. Auditory Verbal Learning Test-Immediate Recall (AVLT-IR) and Trail Making Test-B (TMT-B) were used to assess episodic memory and executive function, respectively.

### MRI acquisition and analysis

High-resolution 3D T1-weighted (TR = 8.5ms, TE = 3.2ms, flip angle = 12, voxel size = 1 × 1 × 1 mm^3^) and T2-weighted FLAIR images (TR = 8500ms, TE=137ms, flip angle = 12; voxel size = 1 × 1 × 1 mm^3^) were acquired on a GE Signa 3T time-of-flight (TOF) PET-MR (GE Healthcare, Milwaukee, WI, USA) in 39 HC and 33 LLD participants. Multishell diffusion MRI data were acquired in 30 HC and 23 LLD participants with the following parameters: 12, 20, 32, and 60 gradient directions at *b*-values of 0, 700, 1000, and 2000 s/mm^2^, respectively, voxel size = 2.5 × 2.5 × 2.5 mm^3^, TR = 6.6 s, TE = 87.4ms, matrix size 96 × 96, multiband factor = 2, phase-encoding direction = A-P. A second scan was also acquired with a reverse-phase encoding and 11 directions (5 × *b* = 0 and 6 × *b* = 2000 s/mm^2^) for correction of susceptibility-induced distortions.

All T1-weighted images were processed using the default pipeline of the Computational Anatomy Toolbox (CAT12, v12.6-rc-1). MTL volumes were calculated by summing volumes of the hippocampus and amygdala defined by the Neuromorphometrics Atlas. Total intracranial volume (TIV) was calculated using CAT12. Regional brain volumes were normalized by regressing out the effect of TIV. A segmentation of FLAIR images was conducted using icobrain (Rakić et al., 2021) to calculate WMH volumes. All segmentation results of FLAIR images were visually checked and manually corrected by a licensed neurologist (MVC).

Diffusion-weighted images were pre-processed using in-house analysis pipeline (https://github.com/treanus/KUL_NIS) and publicly available software, including FSL (https://fsl.fmrib.ox.ac.uk/fsl/fslwiki) and MRtrix3 (https://www.mrtrix.org/). Pre-processing pipeline includes denoising, removal of Gibbs ringing artifacts, motion and eddy current correction, bias field correction, and distortion correction using reverse phase-encoding images. A diffusion kurtosis tensor model was fit using DIPY (https://dipy.org/index.html) to calculate mean kurtosis (MK), axial kurtosis (AK), and radial kurtosis (RK). A diffusion tensor model was fit using MRtrix3 to generate FA and mean diffusivity (MD) maps. We performed bundle-specific analysis of diffusion metrics in the following MTL-related bundles reconstructed using CSD tractography implemented in the KUL_FWT pipeline (https://github.com/KUL-Radneuron/KUL_FWT) (Radwan et al., 2022): uncinate fasciculus, fornix, and cingulum bundle. In our analysis pipeline, the cingulum bundle is divided into dorsal (cingulate) and ventral (temporal) cingulum. While only the temporal cingulum directly connects to the MTL, both segments were analyzed in this study as the dorsal cingulum is indirectly connected to the MTL and is relevant in depression (Alagapan et al., 2023; Wen et al., 2014).

### PET acquisition and analysis

A 30-min ^18^F-MK-6240 PET-MR (p-tau) scan, acquired 90–120 min postinjection was obtained in 39 HC and 19 LLD participants using a GE Signa 3T TOF PET-MR. The MR images were acquired simultaneously. All PET images were processed with an in-house MATLAB script (https://github.com/THOMVDC/PSYPET) to obtain standardized uptake value ratio (SUVR) images, using cerebellar GM as a reference region. Partial volume correction was performed with a validated in-house region-based voxel-wise partial volume correction (RBV PVC) algorithm (Mertens et al., 2022).

### Statistical analysis

Analysis of clinical characteristics, descriptive statistics, and group comparisons were performed using R (version 4.2.2.). Distributions of all variables were inspected using histograms and Shapiro–Wilk tests. Group comparisons were conducted using a two-sample *t* test, Mann–Whitney *U* test, Chi-squared test, or Fisher’s exact test, depending on the data distribution.

First, to investigate GM and WM differences, group comparisons were conducted between patients with LLD and healthy subjects. GM differences were assessed by comparing MTL volumes between the groups using a two-sample *t* test with a statistically significant threshold set at *p* < 0.025 (corrected for the two MTL volumes, i.e., left and right hemisphere). WM differences were examined through voxel-based analyses (VBAs) of MTL-related WM tracts, including the uncinate fasciculus, cingulum bundle, and fornix. MK was the primary outcome measure for WM analysis, with other DKI-derived metrics (i.e., AK and RK), and DTI-derived metrics (i.e., FA and MD) as secondary outcomes. The statistical threshold for WM analyses was set at a family-wise error (FWE) corrected *p* < 0.05, determined by threshold-free cluster enhancement (TFCE). The group comparisons of DKI/DTI metrics were performed using FSL’s randomize.

Second, to investigate aetiological factors contributing to GM and WM differences in LLD, two multiple regression analyses were performed. Dependent variables in each model included GM or WM differences detected in the group comparisons. Independent variables included the SUVR of ^18^F-MK-6240 in the MTL and WMH volumes as proxies for a pathological aging process (e.g., increased tau accumulation) and a cerebrovascular-related brain aging process (e.g., increased WMH volumes), respectively. To mitigate the effects of multicollinearity and multiple comparisons, the following analyses were conducted. Bilateral MTL volumes were averaged, and this mean was used as a measure of ‘GM differences’. Additionally, principal component analysis (PCA) was applied for the DKI-/DTI-metrics of each WM bundle, which showed group differences. The first principal component score was used as a summary measure of ‘WM differences’ and subsequently used as an independent variable in the models mentioned above. WMH volumes were log-transformed as they were positively skewed. Before entering multiple regression models, the age effect was regressed out of all variables (i.e., we conducted regression analyses including age as a predictor, and the residuals were extracted). Additionally, those residuals were scaled to have a mean of 0 and a standard deviation of 1 to use all values with different scales in the multiple regression models.

Third, to investigate the potential impact of clinical characteristics of LLD, such as onset age and duration of the current episode, on GM and WM differences, correlation analyses were conducted with a statistically significant threshold of *p* < 0.05. As an additional exploratory analysis, we investigated potential relationships between brain tissue differences and clinical symptoms (i.e., GDS and MADRS total scores) as well as cognitive function (i.e., MMSE, AVLT-IR, and TMT-B).

## Results

A total of 72 participants were included: 33 subjects were patients with LLD and 39 were HC (Table 1). There were no statistically significant differences in mean age (*t* = −1.1, df = 69, *p* = 0.27) or sex (χ^2^ = 0.40, df = 1, *p* = 0.47) between the groups. Other clinical characteristics are presented in Table 1.

**Table 1.** Clinical characteristics of the participants

	HC	LLD		*p*-value
*N*	39	32		
Age	70.6 (6.2)	72.1 (5.9)	*t* = –1.1	0.29
Female	24/39 (61.5%)	22/32 (68.8%)	χ^2^ = 0.40	0.53
Psychotic features		10/31 (32.3%)		
Onset age		51.5 (17.7)		
Episode duration, months		19.0 (8.0–52.0)		
GDS	2.6 (2.9)	21.3 (6.8)	*t* = 15.5	<0.001
MADRS	0.92 (1.7)	28.2 (10.5)	*t* = –16.0	<0.001
MMSE	29.0 (1.2)	25.8 (2.9)	*t* = 6.1	<0.001
AVLT-IR	40.8 (10.0)	25.4 (9.9)	*t* = 6.7	<0.001
TMT-B	100.8 (55.9)	232.7 (80.2)	*t* = –7.9	<0.001
Medication				
Antidepressants	0	29/31 (93.5%)		<0.001
Antipsychotics	0	20/31 (64.5%)		<0.001
Benzodiazepines	3/39 (15.8%)	14/31 (45.2%)		<0.001
Anticholinergics	0	3/31 (9.7%)		0.08
ApoE genotype				0.67
ε4 carrier	6/39 (15.4%)	4/30 (13.3%)		

Each variable is described as mean (SD) for continuous variable. Episode duration is described as median (IQR) due to non-normal distribution.

GDS, Geriatric Depression Scale; MADRS, Montgomery-Asberg Depression Rating Scale, MMSE, Mini-Mental State Examination; AVLT-IR, Auditory Verbal Learning Test-Immediate Recall; TMT-B, Trail Making Test-B

### Assessment of cerebrovascular burden

Normalized WMH volumes were significantly higher in patients with LLD compared with HC [median volumes in LLD and HC were 4.05 × 10^3^ mm^3^ (IQR = 2.26–10.3 ×10^3^) and 2.71 × 10^3^ mm^3^ (IQR = 0.92–5.10 × 10^3^), *p* = 0.03]. When distinguishing periventricular and deep WMH, group differences were observed only in the periventricular (*p* = 0.015) but not in deep WMH (*p* = 0.32).

### Group comparison of MTL volumes and MTL-related WM tracts

**Figure 1.**
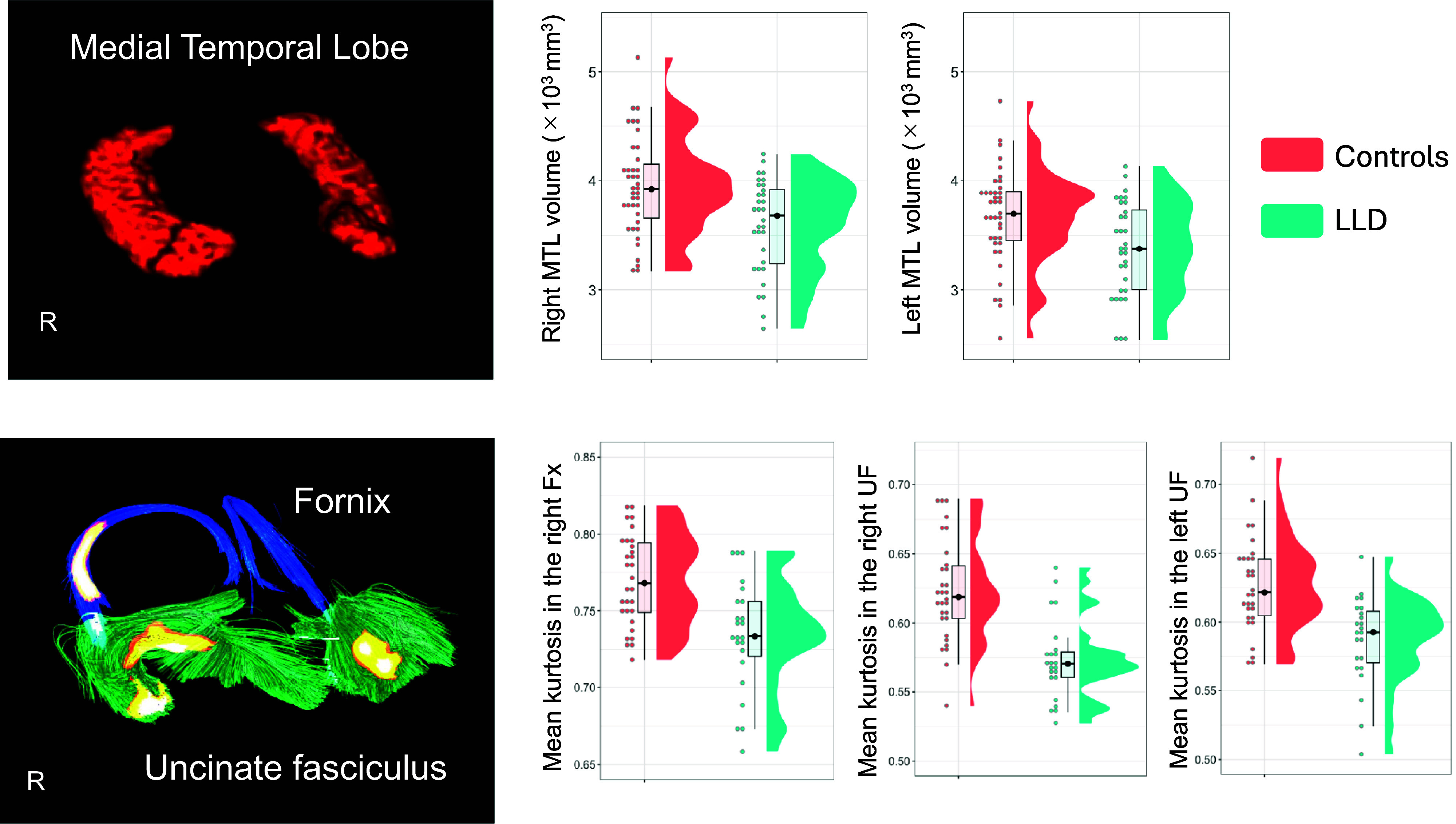
Group comparisons of (A) MTL volumes and (B) MTL-related fiber bundles. (A) Patients with LLD (turquoise) showed significantly lower MTL volumes bilaterally compared to healthy controls (red). (B) They also showed significantly lower MK in the bilateral uncinate fasciculi and the right fornix.

Compared with HC, patients with LLD showed significantly lower right (*t* = 3.4, df = 70, *p* = 0.001; Cohen’s *d* = 0.80) and left (*t* = 2.9, df = 70, *p* = 0.005; Cohen’s *d* = 0.69) MTL volumes (Figure 1A).

Compared with HC, patients with LLD showed significantly lower MK in the bilateral uncinate fasciculi and right fornix (FWE-corrected *p* < 0.05 determined by TFCE) (Figure 1B). Results of other DKI metrics (i.e., AK and RK) and DTI metrics (i.e., MD and FA) are presented in supplementary material (Supplementary Figure 1–5).

### Effects of cerebrovascular and neurodegenerative processes on GM/WM differences

The first principal component, which explained 46.0% of the variance of DKI/DTI metrics in patients with LLD, was used in the following analyses (Supplementary Figure 6).

**Figure 2.**
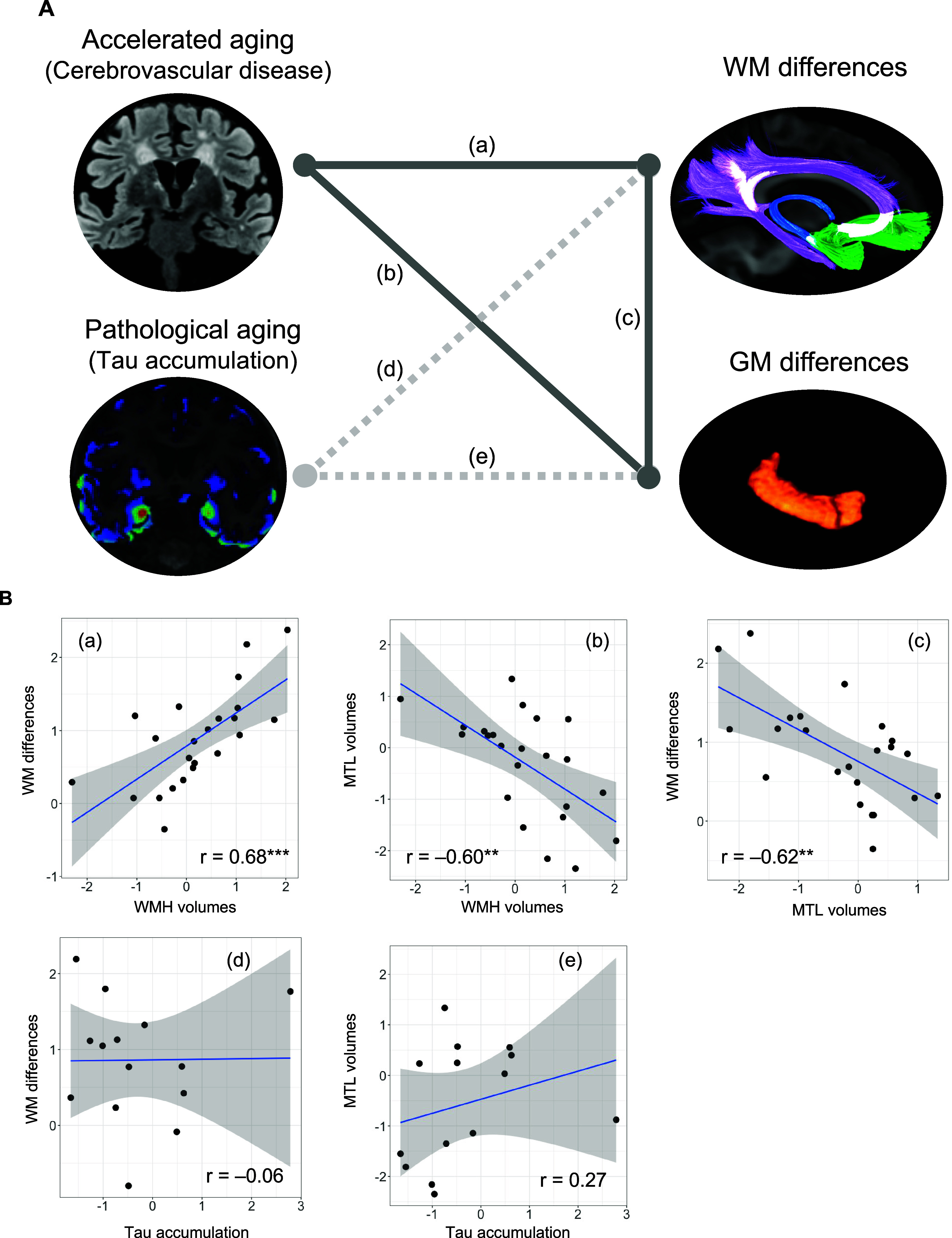
Associations between aetiological factors and brain tissue differences in patients with LLD. (A) Multiple regression models showed that cerebrovascular burden was associated with both GM and WM differences, while tau accumulation in the MTL was not associated with them. (B) To illustrate the associations between the factors, scatter plots are presented. All variables are normalized, and age is regressed out. Cerebrovascular burden was significantly correlated with (a) WM differences (*r* = 0.68, *p* < 0.001) and (b) GM differences (*r* = –0.60, *p* = 0.003). (c) There was a significant correlation between WM and GM differences (*r* = –0.62, *p* = 0.002). Tau accumulation was not associated with (d) WM differences (*r* = –0.06, *p* = 0.85) or (e) GM differences (*r* = 0.27, *p* = 0.34). ***p < 0.001, **p < 0.01, *p < 0.05.

In the multiple regression models, cerebrovascular burden was significantly associated with both MTL volume (Standardized Beta = –0.82, SE = 0.29, *p* = 0.015) and WM differences (Standardized Beta = 0.62, SE = 0.17, *p* = 0.004), while AD-type tau burden was not associated with MTL volume (Standardized Beta = 0.36, SE = 0.22, *p* = 0.14) or WM differences (Standardized Beta = –0.1, SE = 0.13, *p* = 0.49) (Table 2; Figure 2). MTL volume and WM differences were significantly inversely correlated (*r* = –0.62, *p* = 0.002).

**Table 2.** Aetiological factors associated with brain tissue differences

	Beta	SE	*p*-value
Model 1: GM differences			
Cerebrovascular burden	–0.82	0.29	0.015
Tau accumulation	0.36	0.22	0.14
Model 2: WM differences			
Cerebrovascular burden	0.62	0.17	0.004
Tau accumulation	–0.1	0.13	0.49

### Association between clinical characteristics of LLD and GM/WM differences

Duration of the current depressive episode was negatively correlated with MTL volumes (*r* = –0.56, *p* = 0.01) but not with WM differences (*r* = 0.13, *p* = 0.59). Onset age was not correlated with MTL volumes or WM differences. None of the clinical symptoms or cognitive function showed significant correlations with the GM/WM differences (Supplementary Figure 7).

## Discussion

Our multimodal PET-MRI study yielded the following key findings: (1) lower GM volumes in the MTL and microstructural differences in MTL-related WM bundles, mainly the uncinate fasciculus and fornix, in patients with LLD; (2) a significant association between GM/WM differences and cerebrovascular burden in LLD; (3) a lack of association between GM/WM differences and tau accumulation in LLD; (4) a significant association between GM and WM differences in LLD; and (5) a significant association between the duration of the current depressive episode and lower GM volumes in the MTL. These results suggest that higher cerebrovascular burden may play a more significant role in the pathophysiology of brain tissue differences in LLD than the pathological aging process of AD-type tau accumulation. Additionally, depressive episodes themselves may be associated with GM differences in patients with LLD.

While numerous studies have reported brain tissue differences in LLD (Kim & Han, 2021), only a few studies simultaneously investigated both GM and WM in the same subjects (Harada et al., 2016; Sexton et al., 2012). However, these studies, using T1 structural MRI to assess GM and DTI to assess WM, have reported mixed results: one study reported no GM differences with lower FA in widespread brain regions (Sexton et al., 2012), while another showed lower GM in frontolimbic regions accompanied by higher FA in the uncinate fasciculus (Harada et al., 2016). In contrast to these earlier studies, we focused on the MTL and its connecting WM tracts, given their critical role in LLD, as well as their significance in emotional and cognitive functions (Geerlings and Gerristen, 2017; Kim & Han, 2021; Wen et al., 2014). Furthermore, we utilized advanced diffusion MRI modeling for the first time in an LLD cohort to account for non-Gaussian diffusion using DKI and minimize the confounding effects of crossing fibers on tract reconstruction using CSD, thereby enhancing the reliability of our findings compared to those derived from DTI modeling. Our finding of higher, not lower, FA in the uncinate fasciculus may be counterintuitive. However, the higher FA is in line with a previous study (Harada et al., 2016) and could be driven by lower fiber dispersion compared to controls.

Notably, our finding of a positive correlation between MTL GM volume and WM metrics sheds new light on the neurobiology of LLD. This finding suggests three potential scenarios: (1) GM abnormalities lead to altered WM microstructure; (2) altered WM microstructure leads to GM volume reduction; and (3) a shared aetiological factor mediates differences in both GM and WM. The concept of WM abnormalities as a secondary phenomenon is consistent with Wallerian degeneration, where WM differences follow GM cell loss. Although definitive conclusions about the temporal sequence of GM and WM abnormalities require longitudinal studies, secondary WM degeneration has been characterized by reduced axial diffusivity with increased radial diffusivity (Pierpaoli et al., 2001; Song et al., 2002), which differs from our findings of reduced axial and radial kurtosis in patients with LLD. Another explanation for the GM–WM relationship is that WM dysconnectivity impedes the normal transneuronal transport of trophic factors, leading to GM atrophy. This atrophy might not necessarily reflect cell loss but could reflect dendritic shrinkage and/or reduced synaptic density, although a recent study did not find any evidence of lower synaptic density in patients with LLD (Vande Casteele et al., 2022). For example, the axonal transport of cholinergic inputs from the basal forebrain to the hippocampus via the fornix facilitates the production of brain-derived neurotrophic factor (BDNF), fostering the growth, and proliferation of neurons in the hippocampus (Erickson et al., 2010; Murer et al., 2001).

In this study, we examined the contribution of different neurobiological phenomena associated with brain aging to LLD, making the distinction between ‘pathological’ aging related to increased amyloid and/or tau accumulation and cerebrovascular-related brain aging, which may be more pronounced or ‘accelerated’ in LLD (Szymkowicz et al., 2023). In neurodegenerative diseases, such as AD, abnormal protein aggregation causes cell loss and subsequently GM atrophy. However, increasing evidence suggests LLD may not be associated with clinically significant amyloid burden (De Winter et al., 2017; Mackin et al., 2021; Takamiya et al., 2021) or tau (Vande Casteele et al., 2022). In addition, we found no correlation between tau accumulation in the MTL and GM/WM differences in LLD. While subtle changes in protein homeostasis without visible deposits of aggregated pathological proteins might still contribute to GM atrophy in the MTL, this hypothesis requires further research. On the other hand, the ‘accelerated’ aging hypothesis includes various phenomena associated with normal biological aging, including cerebrovascular changes. Our finding of higher WMH in patients with LLD is consistent with the ‘vascular depression hypothesis’ (Taylor et al., 2013) and the results of a systematic review and meta-analysis that reported a link between peripheral and cerebral microvascular dysfunction and LLD (van Agtmaal et al., 2017). In this context, we contributed new insights into the role of cerebrovascular burden on LLD, demonstrating its association with reduced MTL volume and reduced WM integrity. From a clinical point of view, our results suggest that cerebrovascular disease contributes to the increased risk of GM and WM tissue alterations in LLD. Furthermore, it could be hypothesized that preventative treatments for cerebrovascular disease could mitigate/reduce this risk.

An additional factor potentially contributing to tissue differences in LLD, although not directly investigated in our current study, is genetic predisposition, as suggested by previous research. Specifically, the BDNF polymorphism (i.e., Val66Met) has been associated with reduced integrity of the uncinate fasciculus in MDD (Carballedo et al., 2012; Han et al., 2018; Tatham et al., 2017) and with increased WMH volumes in older individuals (Taylor et al., 2009). While the genetic predisposition alone showed weak or no correlation with hippocampal volumes or WM integrity (Pereira et al., 2018), a combination of various generic polymorphisms and gene–environment interactions may contribute to tissue differences in LLD. Furthermore, the influence of cerebrovascular burden appears to be more pronounced in LLD compared to adult MDD, underscoring the need for more focused research within the LLD cohort.

Regarding the associations between brain tissue differences and clinical factors, we found that lower MTL volumes were associated with longer durations of depressive episodes. This result is in line with previous neuroimaging literature in MDD, which reported significant hippocampal volume reduction predominantly in patients with recurrent or longer depressive episodes (McKinnon et al., 2009; Schmaal et al., 2016). The observed GM volume reduction in our study could be a consequence of depressive episodes- or stress-related neurotoxic effects, such as chronic hyperactivity of the hypothalamic–pituitary–adrenal axis, neuroinflammation, or oxidative stress (Belleau et al., 2019). Additionally, animal research has shown stress-induced neuroarchitecture changes, including reduced dendrite and spine density, which may underlie the MRI-detectable brain volume reduction in depressed patients (Sanacora et al., 2022). These insights underscore the clinical need for early intervention to reduce the duration of depressive episodes, alongside efforts to reduce cerebrovascular burden, as a strategy to prevent the progression of brain tissue differences in LLD.

Several limitations of our study should be acknowledged. First, our sample size was modest, although it was comparable to other PET studies in LLD (Madsen et al., 2012; Moriguchi et al., 2021; Wu et al., 2014). Notably, our study represents the first multimodal PET-MRI investigation in LLD cohort, integrating ^18^F-MK-6240 PET imaging, multishell diffusion MRI, FLAIR imaging, and 3D T1-weighted imaging to simultaneously evaluate tau accumulation, WM integrity, WMH, and GM volume. Second, recruiting participants from an academic hospital might have introduced a selection bias toward patients with difficult-to-treat LLD. Consequently, our findings should not be generalized to broader, less severe LLD populations or to individuals with depressive symptoms in community samples without formal diagnoses of depressive disorders. Nonetheless, our results do offer valuable insights into the neurobiology of LLD as a primary psychiatric disorder.

In conclusion, LLD was associated with both lower GM volumes in the MTL and microstructural differences in WM tracts connected to the MTL. These brain tissue differences in LLD were related to cerebrovascular burden but not the neurodegenerative process of AD-type tau accumulation, arguing against tau pathology as a significant underlying pathophysiological mechanism in LLD.

## Supporting information

Takamiya et al. supplementary material Takamiya et al. supplementary material

## Supplementary material

The supplementary material for this article can be found at http://doi.org/10.1017/S0033291724003362.
