# Supplementary material for: Gray and white matter differences in the medial temporal lobe in late-life depression: a multimodal PET-MRI investigation
Source: Psychol Med. 2025 Feb 4;55:e10. doi: 10.1017/S0033291724003362 (PMC11968119; doi:10.1017/S0033291724003362)

Supplementary Figure 1. **Results of voxel-based analysis of mean kurtosis**. For visualization purposes, results are presented in each bundle separately. Compared with healthy controls, patients with late-life depression showed significantly lower mean kurtosis in (A) the right uncinate fasciculus, (B) the right fornix, and (C) the left uncinate fasciculus. The statistical significance threshold was set at FWE-corrected p <0.05 determined by TFCE. The location of significant differences in mean kurtosis between the groups are depicted in red-yellow.

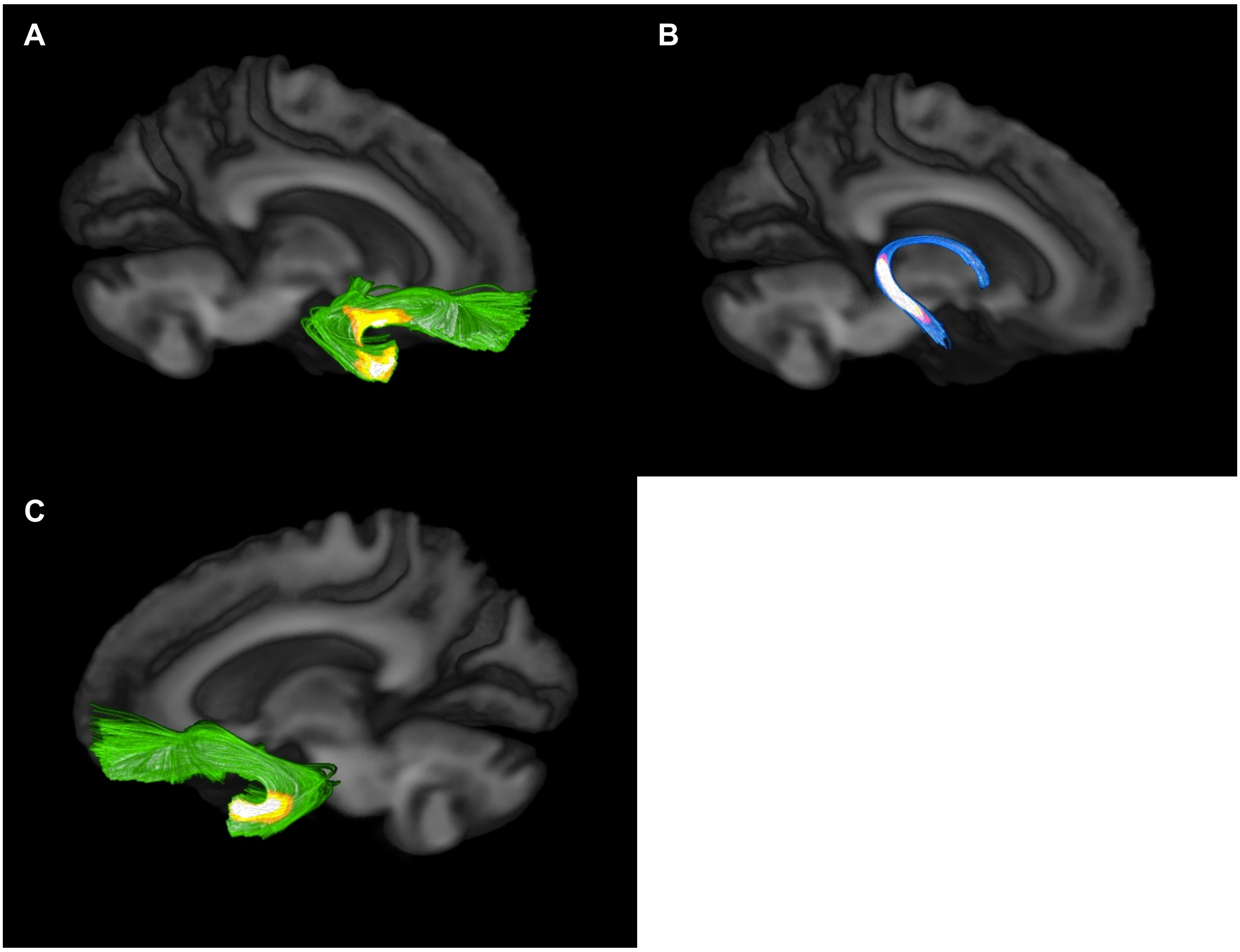

Supplementary Figure 2. Results of voxel-based analysis of radial kurtosis. Compared with healthy controls, patients with late-life depression showed significantly lower radial kurtosis in (A) the right uncinate fasciculus, (B) the right fornix, (C) the left uncinate fasciculus, and (D) the left cingulum bundle. The statistical significance threshold was set at FWE-corrected $p < 0.05$ determined by TFCE. The location of significant differences in radial kurtosis between the groups are depicted in red-yellow. The results of radial kurtosis highly overlapped with the results of mean kurtosis, but the alterations in radial kurtosis were more widespread.

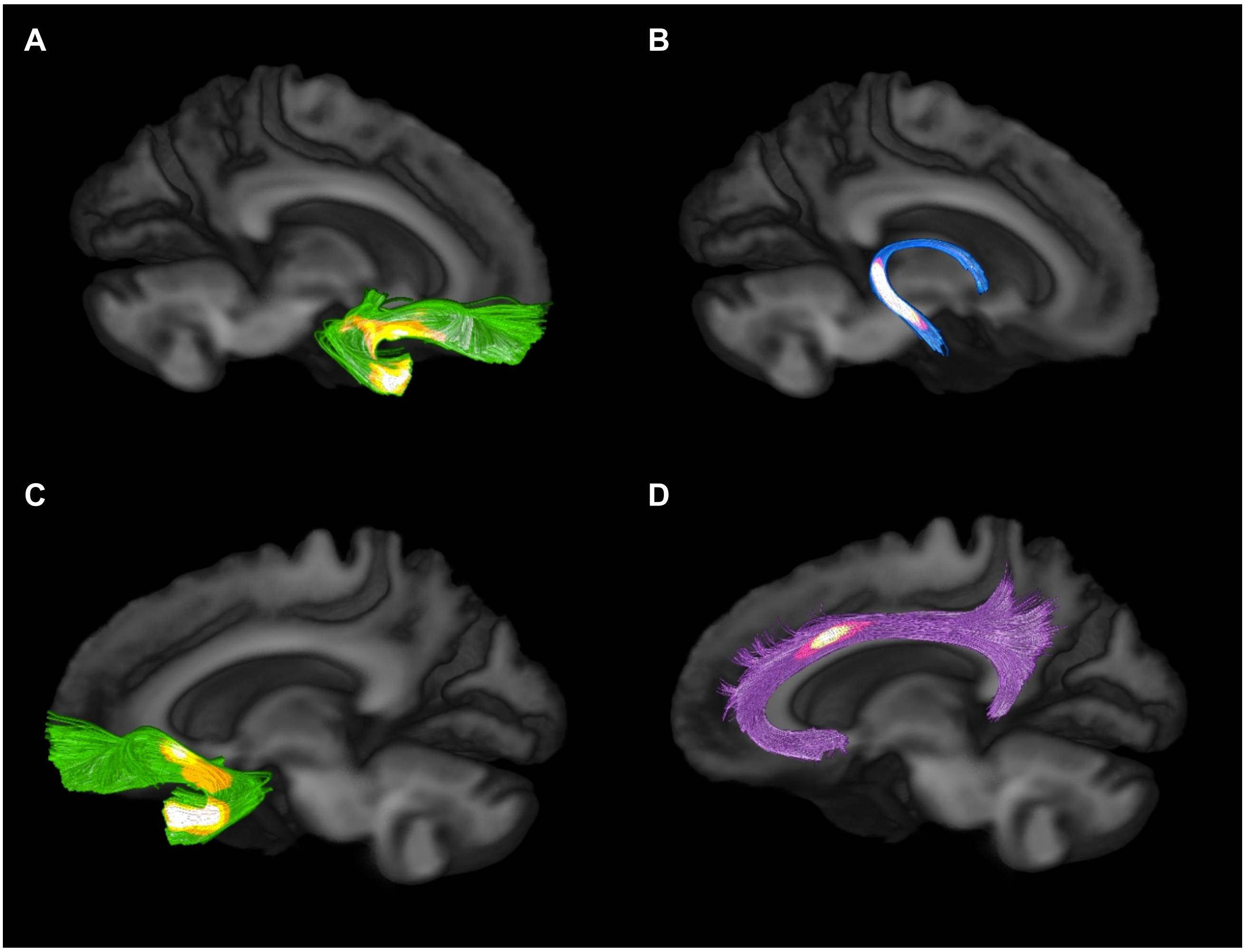

Supplementary Figure 3. Results of voxel-based analysis of axial kurtosis. Compared with healthy controls, patients with late-life depression showed significantly lower axial kurtosis in (A) the right uncinate fasciculus, (B) the right fornix, and (C) the left cingulum bundle. The statistical significance threshold was set at FEW-corrected p <0.05 determined by TFCE. The location of significant differences in axial kurtosis between the groups are depicted in red-yellow.

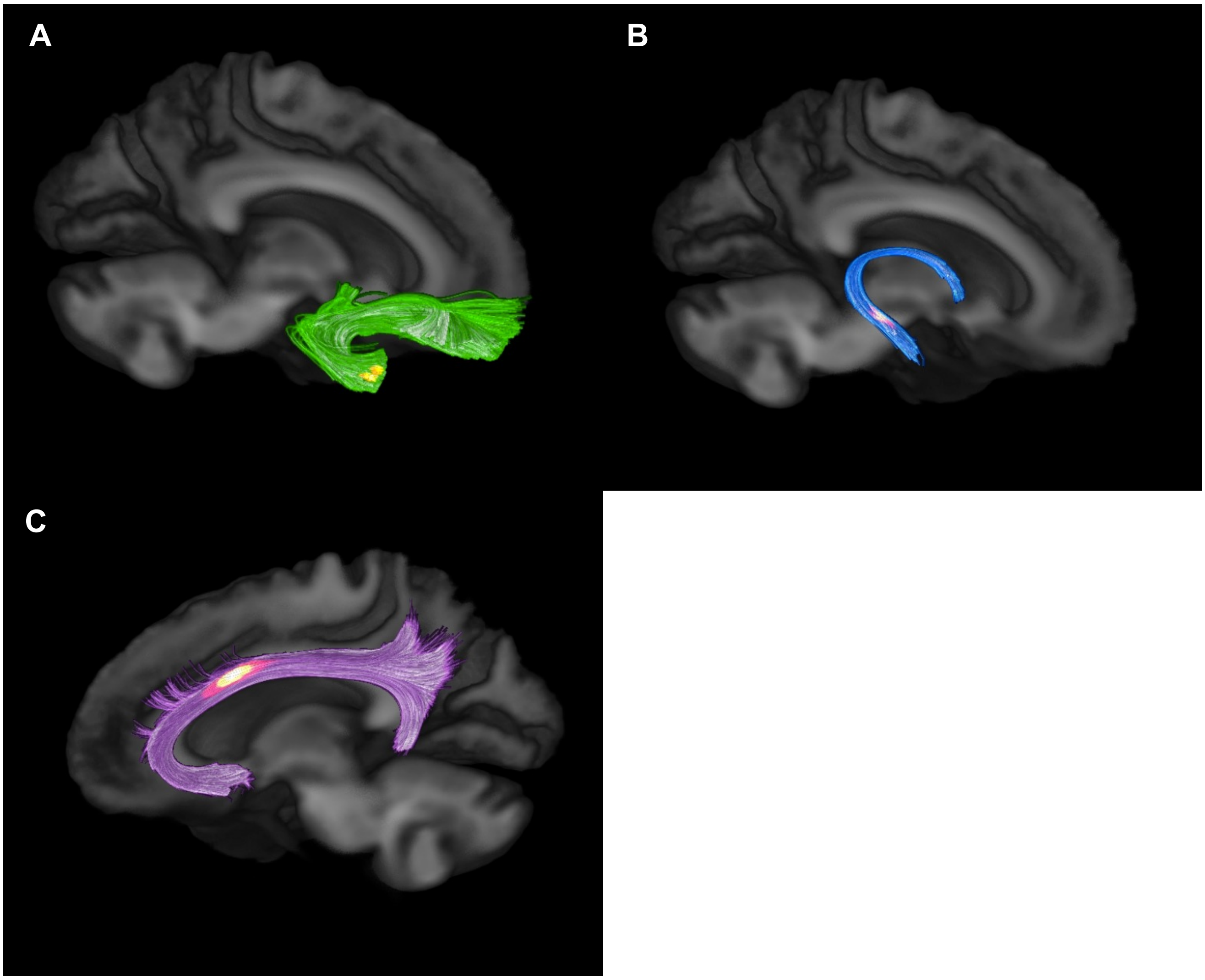

Supplementary Figure 4. Results of voxel-based analysis of mean diffusivity. Compared with healthy controls, patients with late-life depression showed significantly higher mean diffusivity in (A) the right dorsal cingulum, (B) the right ventral cingulum, (C) the left dorsal cingulum, and (D) the left ventral cingulum. The statistical significance threshold was set at FWE-corrected $p < 0.05$ determined by TFCE. The location of significant differences in mean diffusivity between the groups are depicted in red-yellow.

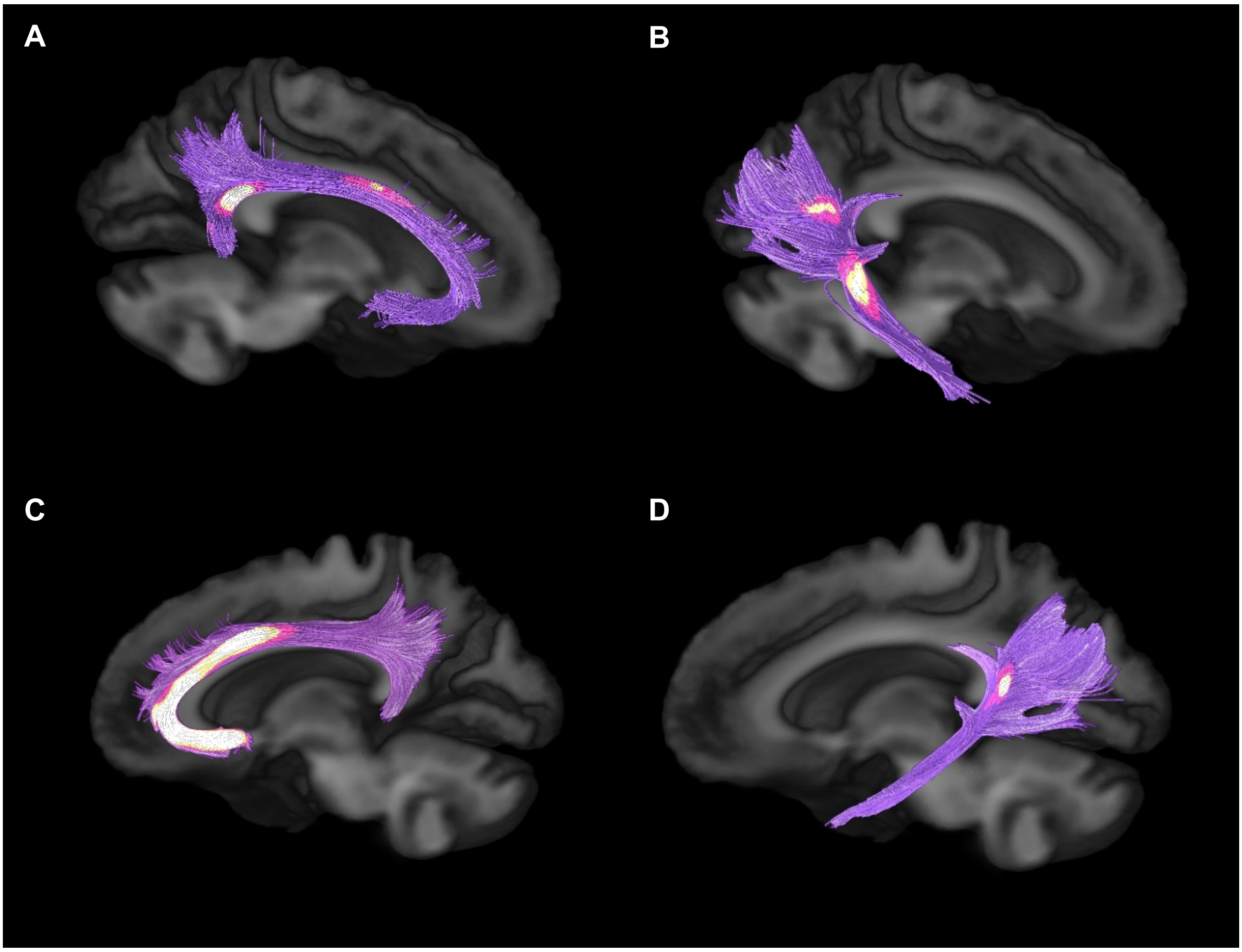

Supplementary Figure 5. Results of voxel-based analysis of fractional anisotropy. Compared with healthy controls, patients with late-life depression showed significantly higher fractional anisotropy in (A) the right uncinate fasciculus, (B) the right ventral cingulum, and (C) the left dorsal cingulum. In contrast, patients with late-life depression they showed lower fractional anisotropy in (D) the left fornix. The statistical significance threshold was set at FWE-corrected $p < 0.05$ determined by TFCE. The location of significant differences in fractional anisotropy between the groups are depicted in red-yellow.

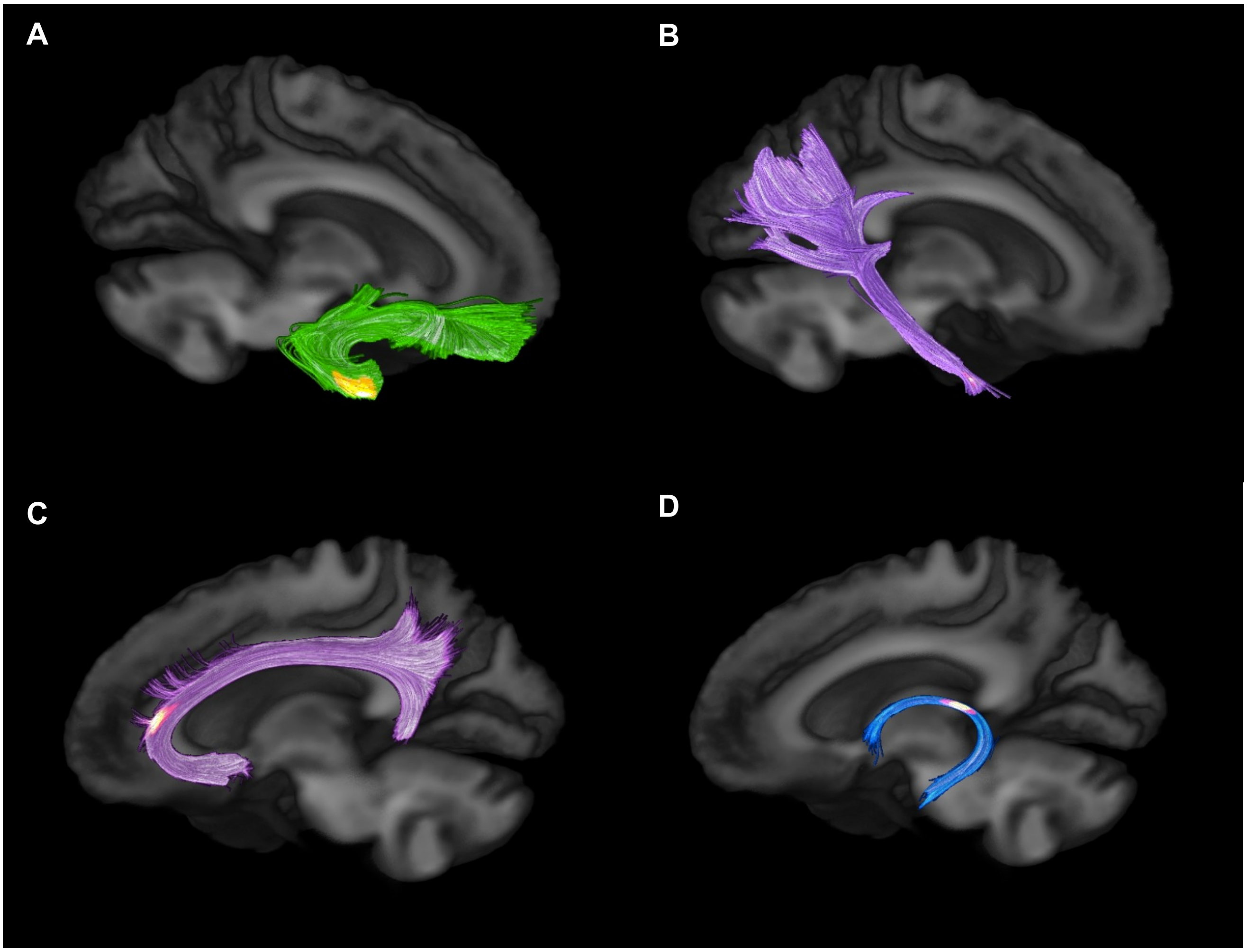

Supplementary Figure 6. Principal component analysis on DKI-DTI-metrics. (A) PCA was performed using “prcomp” function in R. The first principal component (PC1) was used as a proxy for “white matter differences” in patients with LLD. The scree plot shows the PC1 explained a substantial amount (46.0%) of the variance of DKI/DTI-metrics, which showed significant differences in patients with LLD. (B) The percentage of contribution for the PC1 shows that the PC1 captures most aspects (i.e., multiple bundles and multiple metrics) of WM differences in LLD. (C) The loadings of each feature suggest that higher PC1 scores are associated with the LLD-related differences (e.g., increased MD and/or reduced MK).

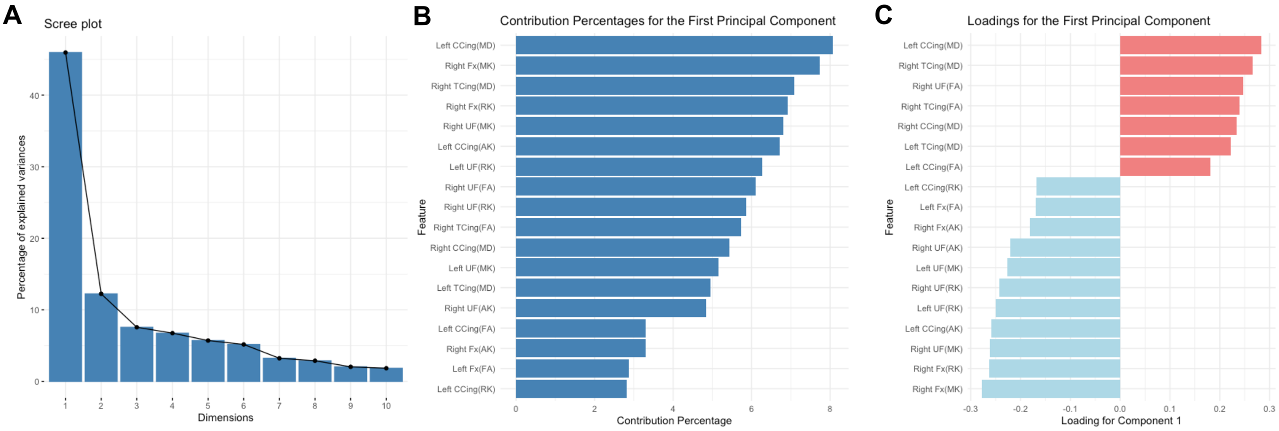

Supplementary Figure 7. Associations between clinical factors and brain tissue differences in patients with LLD. None of the clinical and cognitive measures were associated with brain tissue differences. GDS was not associated with (a) GM difference (r = –0.01, p = 0.96) or (b) WM difference (r = –0.10, p = 0.65). MADRS was not associated with (c) GM difference (r = 0.20, p = 0.35) or (d) WM difference (r = –0.18, p = 0.40). MMSE was not associated with (e) GM difference (r = 0.039, p = 0.86) or (f) WM difference (r = 0.10, p = 0.67). AVLT was not associated with (g) GM difference (r = 0.09, p = 0.75) or (h) WM difference (r = –0.14, p = 0.56). TMT-B was not associated with (i) GM difference (r = –0.21, p = 0.36) or (j) WM difference (r = 0.013, p = 0.95).

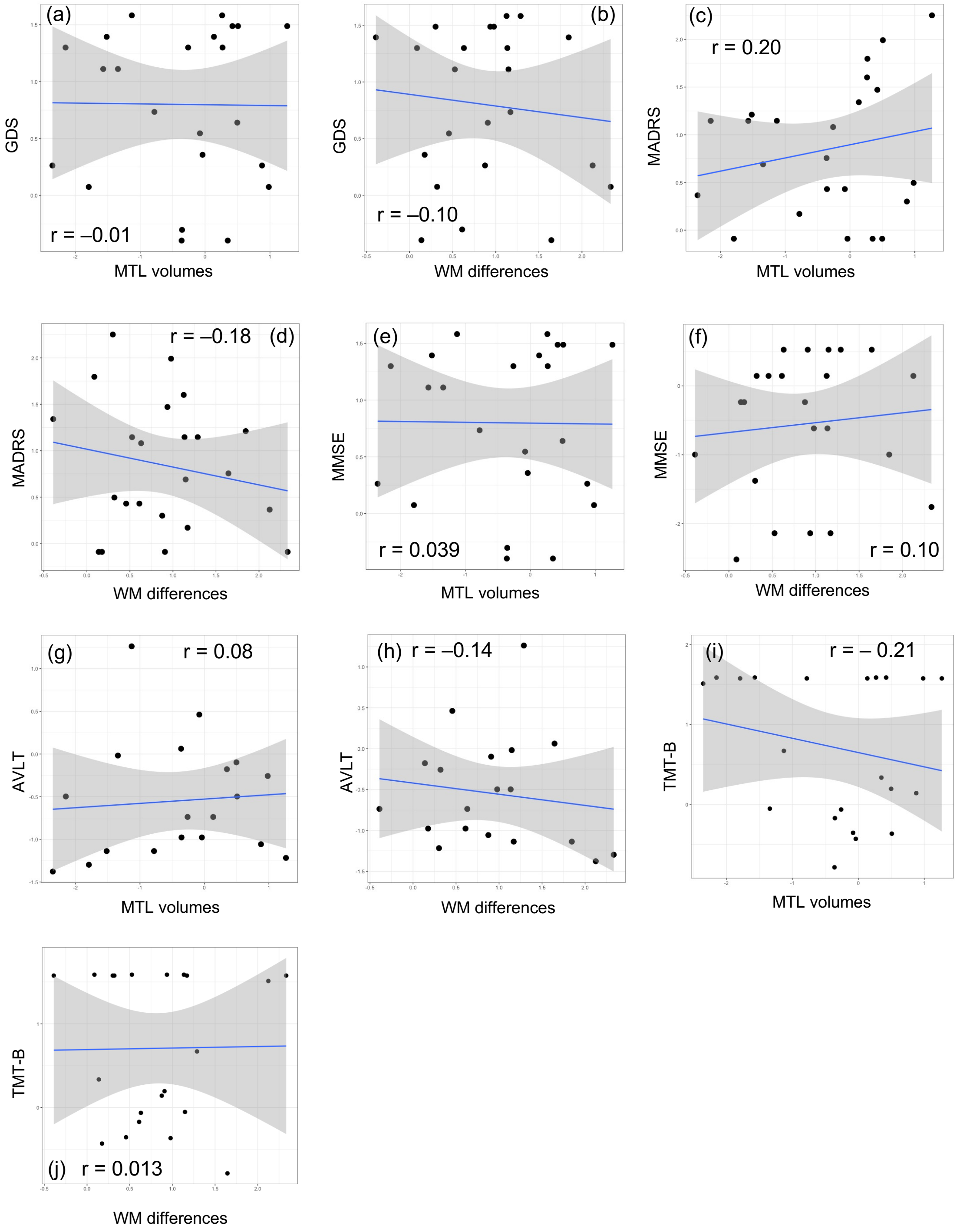

Supplement: Takamiya et al. supplementary material [file S0033291724003362sup001.pdf]
